# Fungicide resistance in *Botrytis cinerea* and identification of *Botrytis* species associated with blueberry in Michigan

**DOI:** 10.3389/fmicb.2024.1425392

**Published:** 2024-07-22

**Authors:** Joel A. Abbey, Safa A. Alzohairy, Kerri A. Neugebauer, Ross J. Hatlen, Timothy D. Miles

**Affiliations:** Department of Plant, Soil, and Microbial Sciences, Michigan State University, East Lansing, MI, United States

**Keywords:** *Vaccinium*, *Botrytis* species, fungicide resistance, blueberry, population genetics

## Abstract

Botrytis blossom blight and fruit rot, caused by *Botrytis cinerea*, is a significant threat to blueberries, potentially resulting in substantial economic losses if not effectively managed. Despite the recommendation of various cultural and chemical practices to control this pathogen, there are widespread reports of fungicide resistance, leading to decreased efficacy. This study aimed to characterize the resistance profile of *B. cinerea* isolated from blighted blossoms and fruit in 2019, 2020 and 2022 (*n* = 131, 40, and 37 for the respective years). Eight fungicides (fludioxonil, thiabendazole, pyraclostrobin, boscalid, fluopyram, fenhexamid, iprodione, and cyprodinil) were tested using conidial germination at specific discriminatory doses. Additionally, 86 isolates were phylogenetically characterized using the internal transcribed spacer regions (*ITS*) and the protein coding genes: glyceraldehyde-3-phosphate dehydrogenase (*G3PDH*), heat-shock protein 60 (*HSP60*), and RNA polymerase II second largest subunit (*RPB2*). This revealed higher fungicide resistance frequencies in 2020 and 2022 compared to 2019. Over all 3 years, over 80% of the isolates were sensitive to fludioxonil, fluopyram, and fenhexamid. Pyraclostrobin and boscalid showed the lowest sensitivity frequencies (<50%). While multi-fungicide resistance was observed in all the years, none of the isolates demonstrated simultaneous resistance to all tested fungicides. *Botrytis cinerea* was the most prevalent species among the isolates (74) with intraspecific diversity detected by the genes. Two isolates were found to be closely related to *B. fabiopsis*, *B. galanthina*, and *B. caroliniana* and 10 isolates appeared to be an undescribed species. This study reports the discovery of a potentially new species sympatric with *B. cinerea* on blueberries in Michigan.

## 1 Introduction

Highbush blueberry (*Vaccinium corymbosum*) production represents a valuable component of the agricultural industry in Michigan and the United States. It is a high-value crop due to its high nutritional benefits (Tobar-Bolaños et al., 2021). In 2022, Michigan produced 26.5 million kg of blueberries across 16,000 acres of cultivated lands with a farm gate price of approximately $96 million. The overall value of blueberry production in Michigan is estimated to be $530 million (USDA, 2023).

Blueberry production is challenged by several diseases, including several viruses, Monilinia blight (mummy berry), Botrytis blight and fruit rot, stem and twig blights, and anthracnose (Retamales and Hancock, 2018; Cline and Burrack, 2023). Botrytis blight and fruit rot are important diseases caused by *Botrytis cinerea*. On blueberries, the fungus commonly infects flowers at the mid to late bloom stage; it can also infect young leaves and shoots. Infected blossoms show symptoms of light brown necrosis, which progresses to dark brown/black as the infected tissues senesce (Smith, 1998). Gray masses of conidia appear on senescent blossoms (Smith, 1998; Abbey et al., 2018). Infection usually occurs when there are several hours to days of wetness and moderate temperatures (14 to 28°C) during bloom (Gossen and Lan, 2021). If not controlled, infections may result in dead blossoms or deformed berries. The fungus can also develop into the ovary and the peduncle and may remain dormant while the fruit is maturing (Molly and Grant-Downton, 2016). These dormant infections may cause fruit rot when the fruit ripens, and berry content and environmental conditions become favorable for fungal growth.

The genus *Botrytis* is both morphologically and genetically diverse, with over 30 species identified (Garfinkel, 2021). While some of these species, such as *B. cinerea*, have a broad host range, other species tend to be host, tissue, and region specific (Molly and Grant-Downton, 2016). *B. cinerea* is described as a species complex with new cryptic species discovered often. *B. cinerea* was initially grouped into Group I and Group II based on the presence or absence of transposons *Boty* and *flipper* (Diolez et al., 1995; Levis et al., 1997). Nonetheless, isolates in Group I were later identified as a new species called *B. pseudocinerea* based on phenotypic and multigene phylogenetic analysis (Walker et al., 2011; Walker, 2016). It is challenging to distinguish *Botrytis* species based on only morphological features, hence several molecular methods, including restriction fragment length polymorphism (RFLP), DNA fingerprinting, and phylogenetic analysis, have been employed to support species delineation and classification (Richards et al., 2021). Phylogenetic analysis of glyceraldehyde-3-phosphate dehydrogenase (*G3PDH*), heat-shock protein 60 (*HSP60*) and DNA-dependent RNA polymerase subunit II (*RPB2*) have been instrumental in *Botrytis* spp. identification and classification (Staats et al., 2005). The analysis of these three genes together with the genes *NEP1* and *NEP2* are the commonly used methods in species delineation in *Botrytis* taxonomy (Staats et al., 2007; Garfinkel, 2021; Richards et al., 2021).

*Botrytis* incited diseases are best managed by fungicide applications and the removal of dead twigs or shoots during the dormant season (Bika et al., 2021; Mundy et al., 2022). No blueberry cultivar is resistant to *B. cinerea*. Hence, the use of fungicides, such as methyl benzimidazole carbamates (Fungicide Resistance Action Committee (FRAC) 1), dicarboximides (FRAC 2), succinate dehydrogenase inhibitors (SDHIs; FRAC 7), anilinopyrimidines (FRAC 9), quinone outside inhibitors (QoIs; FRAC 11), phenylpyrroles (FRAC 12), and hydroxyanilides (FRAC 17), has been employed for *Botrytis* control in blueberry and other crops (Fillinger and Walker, 2016; Alzohairy et al., 2021; FRAC, 2023). Due to the polycyclic nature of the fungus and high fungicide use, reports of resistance development among *B. cinerea* populations are common (Alzohairy et al., 2021; Malandrakis et al., 2022; Wang et al., 2022; Gao et al., 2023). The development of resistance in pathogen populations is of concern, as it can lead to control failures and the application of ineffective fungicides.

Infections by *Botrytis* species are frequent and widespread, especially under wet and warm conditions (Carisse, 2016). Since several new species have been reported on different crops such as blackberry, strawberry and blueberry, across different regions ang countries (Li et al., 2012; Saito et al., 2016a; Dowling et al., 2017; Amiri et al., 2018a), it is important to investigate and understand the diversity that may exist among *Botrytis* isolates in Michigan blueberry fields and their fungicide resistance status. Additionally, it is uncertain if only *B. cinerea* is responsible for both blossom blight and fruit rot or if there may be other *Botrytis* species causing these diseases. Understanding the presence and diversity of *Botrytis* species in Michigan blueberry fields is crucial for assessing the phytopathological threat. This would aid in making appropriate management decisions, since some species can vary in their sensitivity to different fungicides or control products (Fournier et al., 2003).

This research is an extension of previous studies on fungicide resistance among *B. cinerea* isolates from Michigan vineyards (Alzohairy et al., 2021) as there is no such study on *Botrytis* isolates from Michigan blueberry fields. Considering the significant cultural and morphological diversity among *Botrytis* isolates collected from blueberry fields in Michigan, it was important to characterize these isolates to assess their identity. The objectives of this study were therefore to (i) assess the fungicide resistance profile of *B. cinerea* isolates collected from Michigan highbush blueberry fields and (ii) determine whether other *Botrytis* species are present in Michigan.

## 2 Materials and methods

### 2.1 Sample collection and *Botrytis cinerea* isolation

A total of 208 single conidia isolates of *B. cinerea* were collected in 2019, 2020, and 2022 (*n* = 131, 40, and 37 isolates, respectively) from commercial blueberry fields in Michigan. The sampling was conducted in the primary blueberry producing regions of Michigan, Southwest and West Michigan. In 2019 and 2020, isolates were recovered from fruits and in 2022, they were recovered from blossom. Blossom and ripe fruit were sampled from blueberry cultivars commonly grown in Michigan, such as ‘Bluecrop’, ‘Elliot’, ‘Blue Crop’, and ‘Jersey’.

*Botrytis cinerea* conidia from infected samples were spread on water agar media (3% w/v), and the plates were incubated for 16 h at room temperature. Subsequently, isolates were single spored using a sterile needle and transferred onto 20% V8 agar media (100 mL of V8 juice, 1 g of CaCO3, 10 g of agar, and 400 mL of distilled water). For long term isolate storage, isolates were transferred to 1.5% water agar overlaid with 1 cm^2^ sterile glass fiber pieces (Millipore Sigma, United States). After the fungal mycelia had covered the glass fiber pieces, they were placed in sterile coin envelopes and air-dried in magenta boxes in a biosafety cabinet for 48 h. Drierite desiccant (W.A. Hammond, United States) was added to the bottom of the boxes and stored at −20°C (Alzohairy et al., 2021).

### 2.2 Fungicide preparation

Eight fungicides belonging to seven different classes were used in this study. Five technical-grade fungicides; fludioxonil (99.5%) (FRAC 12), thiabendazole (98.6%) (FRAC 1), pyraclostrobin (99.5%) (FRAC 11), boscalid (99.5%), and fluopyram (100%) (FRAC 7) were obtained from Sigma-Aldrich. The remaining three fungicides were commercial products: Elevate 50WDG (50% fenhexamid, FRAC 17, UPL Limited, Mumbai, India), Rovral (41.6% iprodione, FRAC 2, FMC Corporation, Philadelphia, PA) and Vangard (75% cyprodinil, FRAC 9, Syngenta, Basel, Switzerland). Stock solutions of the technical-grade fungicides were prepared in 100% acetone, and the active ingredients from the commercial products were prepared in sterile distilled water. The stock solutions were 1,000 μg/mL, except for fluopyram which was 500 μg/mL. A working solution of 200 μg/mL was prepared for each of the fungicides. All stock solutions were stored in the dark at 4°C.

### 2.3 *Botrytis cinerea* fungicide resistance phenotyping

The sensitivity test was carried out following a modified Weber and Hahn (2011) protocol described by Saito et al. (2016a) and Alzohairy et al. (2021). Sensitivity to fenhexamid, fludioxonil, iprodione, thiabendazole, and pyraclostrobin were assessed on 1% malt extract agar (MEA) media (Sigma Aldrich, India), except with the inclusion of the alternative oxidase inhibitor salicylhydroxamic acid (SHAM) (Sigma Aldrich, United States) at a final concentration of 100 μg/mL for pyraclostrobin (Liang et al., 2015). The sensitivity to boscalid and fluopyram was tested on 0.5% yeast extract agar to prevent sugar interference (Cui et al., 2021). Also, to prevent the interference of amino acids, cyprodinil was assessed on 0.5% sucrose media (Weber and Hahn, 2011). Autoclaved media was cooled to 50°C in a water bath and amended with fungicides. A single discriminatory dose of each fungicide was used as described previously (Weber and Hahn, 2011; Saito et al., 2016a; Alzohairy et al., 2021). Fenhexamid, fluopyram, boscalid, cyprodinil, and thiabendazole were tested at 1.0 μg/mL, whereas iprodione and fludioxonil were tested at 0.1 and 5.0 μg/mL, respectively. The study was carried out on 12-well plates filled with 2 mL of the media. For control isolates, the corresponding media was used without the addition of any fungicide. All prepared 12-well plates were stored at 4°C in Ziploc bags and used within 2 weeks of preparation.

Conidial suspensions were prepared from 7- to 14-day-old cultures grown on 20% unclarified V8 agar media. A conidial suspension was prepared by scraping the culture with a sterile toothpick into 1 mL of sterile 0.01% Tween 20 solution (Sigma Aldrich, France). The suspension was filtered through a double layer of cheesecloth, and conidia were counted with a hemacytometer. The suspension was adjusted to a concentration of 10^5^ conidia/ml and kept on ice. Media-filled 12 well plates (Corning Inc., United States) were air-dried in a biosafety cabinet, and 20 μL of conidial suspension of each isolate was applied to each well with two replications. The plates were air-dried in the biosafety cabinet for 10 min and incubated in the dark for 14–16 h at 20°C. A minimum of 20 conidia were examined for germination and germ tube elongation under a compound microscope using ×10 magnification. An isolate was considered fungicide resistant (R) if the length of its germ tube was >50% relative to the germ tube growth on control media, whereas an isolate was considered sensitive (S) if the length of its germ tube was <50% relative to the control.

Statistical analyses were conducted to determine whether there were differences in fungicide sensitivity between the isolates collected from the study years and regions. Two sample t-test, α = 0.05 and UpSet plots were performed using RStudio statistical software 2023.^1^

### 2.4 DNA extraction, PCR amplification, and gene sequencing

Eighty-six *Botrytis* isolates collected from blossoms and fruit were used in this study for molecular characterization. These isolates were labeled as xxB/F - FZ-Y, where xx = year, B/F = blossom/fruit, FZ = field and number, and Y = isolate number. *Botrytis* isolates were grown on 20% unclarified V8 agar and PDA media, and mycelia from 14-day-old cultures were harvested and lyophilized.

For microscopic studies, sporulating structures from PDA plates were mounted on a slide with distilled water. Observations were made with an Olympus compound microscope BX 53 with an Olympus DP27 camera (Evident Scientific, United States). Conidia were observed and analyzed. Measurements of sclerotia (*n* = 10) were performed using digital caliper (Mitutoyo Corporation, Japan).

Approximately 5 mg of mycelia was ground into a powder using a TissueLyser (Qiagen, Valencia, CA). Genomic DNA extraction was conducted on the powdered mycelia using MagMax Plant DNA Isolation Kit (Thermo Fisher Scientific, Waltham, MA) and processed on the KingFisher Flex purification system (Thermo Fisher Scientific). DNA samples were quantified with Qubit 4 fluorometer (Thermo Fisher Scientific) using Qubit 1X dsDNA HS assay kit (Thermo Fisher Scientific).

To identify *Botrytis* species, the partial sequences of the *ITS* regions, *G3PDH*, *HSP60*, and *RPB2* genes of each isolate were amplified using polymerase chain reaction (PCR) and sequenced using sanger sequencing. The primer combinations used in this amplification were the same as those used by Staats et al. (2005) (Supplementary Table S1). PCR reaction was performed in a final volume of 50 μL containing 10× ThermoPol buffer, 1.25 units of Taq DNA polymerase (New England BioLabs, Ipswich, MA), 10 mM of dNTPs, 10 μM of each primer, and 50 ng of DNA. PCR was conducted using a C1000 Touch thermal cycler (Bio-Rad Laboratories, Hercules, CA) with the following conditions: an initial denaturation at 95°C for 3 min, followed by 40 cycles of 95°C for 30 s, 61°C for 30 s, and 68°C for 2 min, and a final extension at 68°C for 10 min. PCR products were examined on 1% agarose gel stained with GelRed (Sigma-Aldrich, United States) and viewed under UV light using a Molecular Imager GelDoc (Bio-Rad Laboratories). Amplicon sizes were determined against a 1 kb molecular ladder. The PCR products were purified using the QIAquick PCR purification kit (Qiagen Inc. United States). Sanger sequencing was performed in both directions on an ABI 3730 x l 96-capillary DNA sequencer at Research Technology Support Facility’s Genomic Core at Michigan State University. The universal M13 primers were used to sequence *ITS*, *HSP60*, and *RPB2* (Staats et al., 2005), while amplification primers were used to sequence *G3PDH*.

### 2.5 Sequence alignment, molecular, and phylogenetic identification

The DNA sequences were assembled in Geneious Prime v. 2023.2.1 (Biomatters Ltd., Auckland, New Zealand). The consensus sequences of all the samples were blasted against the NCBI GenBank database^2^ to establish their phylogenetic relatives. Besides the isolates used in this study, several *ITS*, *G3PDH*, *HSP60*, and *RPB2* gene sequences of reference isolates representing recognized species of *Botrytis* were retrieved from GenBank for phylogenetic analysis (Supplementary Table S2). Sequences from *Sclerotinia sclerotiorum* and *Monilinia fructigena* were included as outgroups for each tree. Multiple sequence alignments of the sequences from this study and those from GenBank were generated using the Muscle alignment tool in Geneious Prime. A concatenated sequence of the three genes (*G3PDH* + *HSP60* + *RPB2*) was generated for each isolate and species to determine the final identity of the isolates. Gaps in the concatenated sequence were treated as missing data.

Phylogenetic analyses were performed by the Maximum Likelihood (ML) and Maximum Parsimony (MP) methods for each of the three genes and the concatenated sequence. ML analyses were performed using IQ-Tree 2.1.3 (Minh et al., 2020). The best nucleotide substitution model for each alignment was selected based on the Bayesian information criterion (BIC) test, and a BS support of 1,000 was used (Kalyaanamoorthy et al., 2017; Hoang et al., 2018). MP analyses were performed in MEGA 11 (Tamura et al., 2021). The clade support values were calculated using 1,000 bootstrap (BS) replicates. The MP tree was generated using the Subtree-Pruning-Regrafting (SPR) algorithm in MEGA 11, using random addition of sequences.

## 3 Results

### 3.1 Fungicides resistance profile

**Figure 1:**
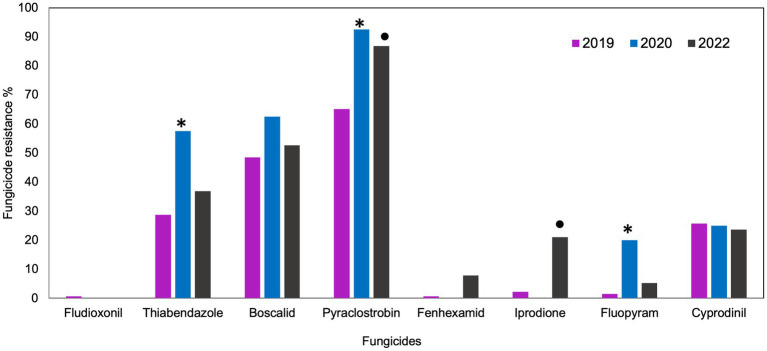
Frequencies of fungicide resistance in *Botrytis cinerea* collected from blossoms and fruit of lueberries grown in Michigan in 2019, 2020 and 2022. The number of isolates was 131, 40 and 37, respectively. Bars with “*” denote a significantly higher (*p* < 0.05) resistance frequency between 2019 and 2020. Bars with “**•**” denote significantly higher (p < 0.05) resistance frequency between isolates collected from fruit (2019 and 2020) and isolated from blossom (2022).

In 2019 and 2020, the outcomes of the discriminatory dose assays indicated different responses to various fungicides. In 2019, 99% of *B. cinerea* isolates were sensitive to fludioxonil and fenhexamid and 98% were sensitive to iprodione and fluopyram (Figure 1). In the same year, 74, 71, 52, and 35% of all isolates were sensitive to cyprodinil, thiabendazole, boscalid, and pyraclostrobin, respectively. In 2020, all the isolates were sensitive to fludioxonil, fenhexamid, and iprodione, whereas 80, 75, 43, 38, and 8% were sensitive to fluopyram, cyprodinil, thiabendazole, boscalid, and pyraclostrobin. In 2022, all the isolates were sensitive to fludioxonil, whereas 95, 92, 78, 75, 62, 46, and 11% were sensitive to fluopyram, fenhexamid, iprodione, cyprodinil, thiabendazole, boscalid, and pyraclostrobin, respectively (Figure 1). Significantly higher frequencies of resistant isolates were detected for thiabendazole (*p* = 0.0001), pyraclostrobin (*p* = 0.000), and fluopyram (*p* = 0.007) in 2020 compared to 2019. Frequencies of resistant isolates from fruit samples (2019 and 2020) were significantly lower for pyraclostrobin (*p* = 0.007) and iprodione (*p* = 0.007) than those of blossom (2022).

The fungicide sensitivity profile of isolates was assessed on a regional basis (Southwest and West Michigan). In 2019, significantly higher frequencies of resistant isolates were observed from the Southwest compared to the West for boscalid (*p* = 0.019) and thiabendazole (*p* = 0.029) (Supplementary Figure S1). In 2020, no resistant isolate was observed for fludioxonil, fenhexamid, and iprodione at both locations. Unlike 2019, there were no significant differences in resistant frequencies of isolates between the two regions. Isolates from the Southwest for all years had higher resistance frequencies for pyraclostrobin (*p* = 0.015), boscalid (*p* = 0.048), and iprodione (*p* = 0.001; Supplementary Figure S1).

**Figure 2:**
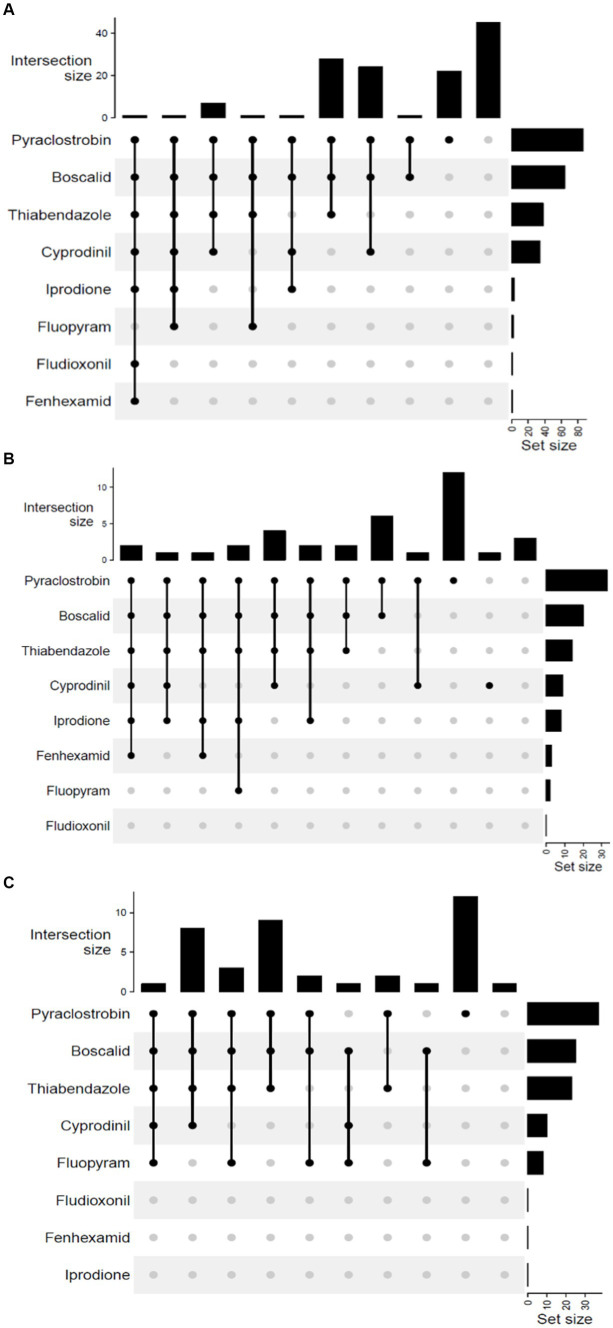
UpSet plot of interactions among the eight fungicides: A summary of the multi-fungicide resistance (MFR) profile in *Botrytis cinerea* across the study years. These plots depict various combinations of MFR observed in **(A)** 2019, **(B)** 2020, and **(C)** 2022. Set size indicates the total number of each fungicide’s representation within the resistance profile. Intersection size indicates the number of isolates resistant to either a single fungicide or a specific set of fungicides (phenotypes). Connected black circles in each plot indicate combinations of fungicides to which one or more isolates show resistance.

Multi-fungicide resistance (MFR) was observed among *B. cinerea* populations in all 3 years. The distribution analysis revealed shared and unique resistance among *B. cinerea* populations, where all isolates were grouped into 10 phenotypes in 2019 and 2020, and 12 phenotypes in 2022 (Figure 2). Only two phenotypes (MFR to pyraclostrobin, boscalid, thiabendazole and cyprodinil and MFR to pyraclostrobin, boscalid, and thiabendazole) were commonly observed in *B. cinerea* populations in all 3 years. None of the *B. cinerea* isolates were simultaneously resistant to all eight fungicides in all years. In 2019, the maximum number of MFR was seven, indicating resistance to all the fungicides except fluopyram, whereas in 2020, a shared resistance of five was the maximum MFR that involved pyraclostrobin, boscalid, thiabendazole, cyprodinil, and fluopyram (Figures 2A,B). In 2022, the maximum number of MFR was six, involving all fungicides except fluopyram and fludioxonil (Figure 2C). The combination of pyraclostrobin, boscalid, and thiabendazole was the most predominant MFR phenotype, constituting 21.4 and 22.5% of the total isolates collected in 2019 and 2020, respectively. The most predominant MFR combination of pyraclostrobin and boscalid accounted for 16.2% of the isolates collected in 2022. In 2019 and 2020, 16.8 and 30% of isolates were resistant (unique) to only pyraclostrobin respectively, whereas 32.4% were unique to only pyraclostrobin in 2022. In 2019, 34.3% of the population was sensitive to all the tested fungicides, compared to 2.5% in 2020. In 2022, 8.1% of the population was sensitive to all the tested fungicides (data not shown).

### 3.2 Fungal identification

This study was conducted with isolates collected in 2019, 2020 and 2022. A total of 86 isolates from fruit (*n* = 37) and blossoms (*n* = 49) were used. The resulting fungal cultures exhibited the typical characteristics of *Botrytis* species. They were initially white, then became grayish to brown after 1 week. Microscopic analysis of sporulating isolates showed oval to elliptical shaped conidia, however not all isolates produced conidia. All the isolates except one produced black sclerotia of varying sizes and shapes after 2 weeks on PDA media at 22°C in the dark. The morphological features of these isolates suggested that they belonged to the genus *Botrytis*.

#### 3.2.1 Morphological characterization of an undescribed *Botrytis* species

**Figure 3:**
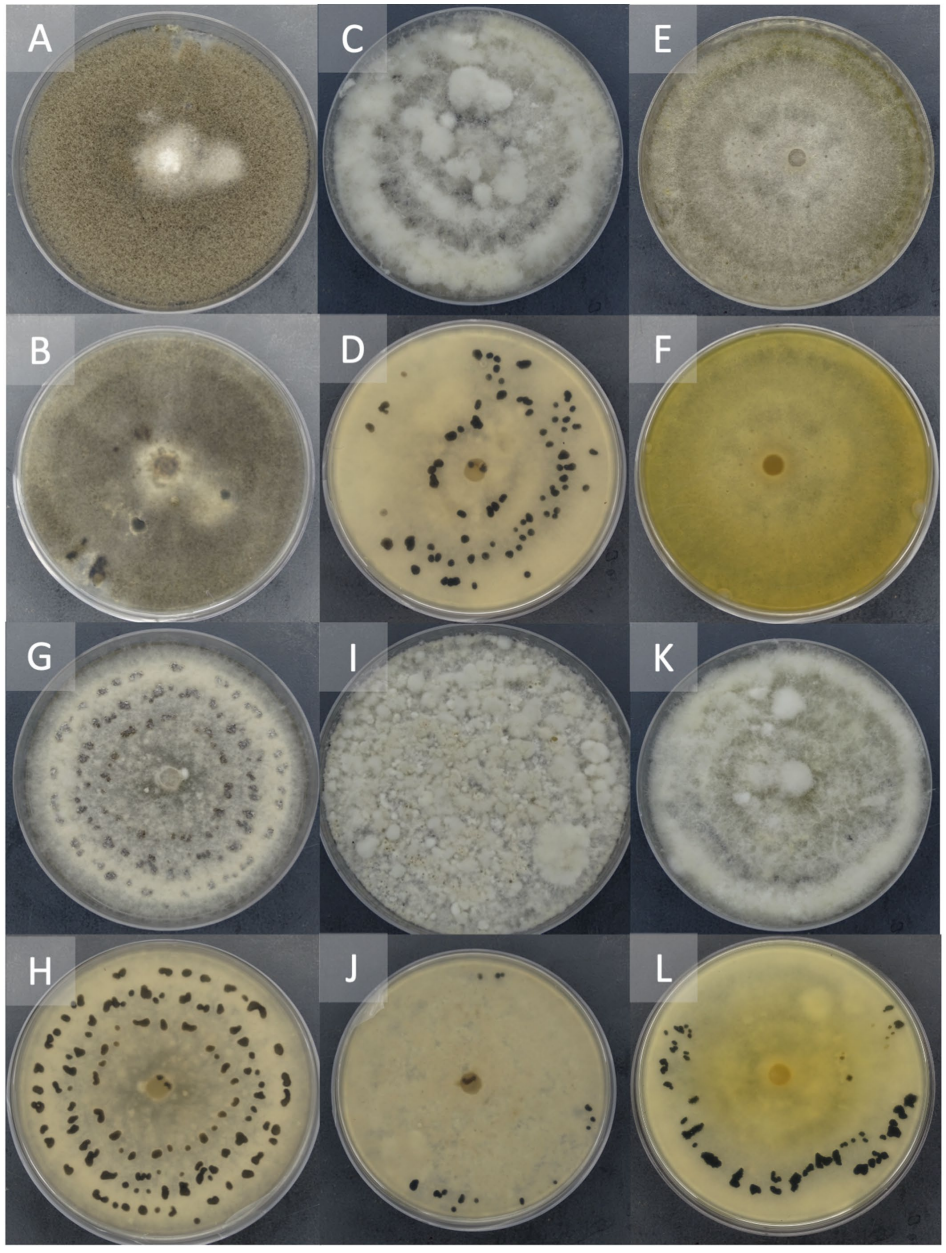
Representation of different morphological characteristics of *Botrytis* species. Two-week-old cultures plated on potato dextrose agar incubated at 22°C in the dark. Front **(A)** and reverse **(B)** of plate of *B. cinerea*, Front **(C)** and the reverse **(D)** of plate of B. sp. 22B-F6-9, Front **(E)** and reverse **(F)** of plate of B. sp. 22B-F11-1, Front **(G)** and reverse **(H)** of plate of B. sp. 22B-F8-7, Front **(I)** and reverse **(J)** of plate of B. sp. 22B-F10-3, Front **(K)** and reverse **(L)** of plate of B. sp. 22B-F7-4.

The isolates of the undescribed *Botrytis* species on PDA were white to pale gray, fluffy, matted, and tufted aerial mycelium. These isolates did not produce conidia in culture but produced black sclerotia in different patterns. Sclerotia were solitary to aggregated, gray to black, irregular, spherical to elliptical with varying sizes (Table 1). While some produced sclerotia in concentric rings, others were randomly formed (Figure 3).

**Table 1:** Sclerotia size of *Botrytis* species after 2 weeks of growth on potato dextrose agar.

Isolate	Sclerotia size (mm)
*B. cinerea*	2.01–3.40 × 2.01–4.80
*B.* sp. 22B-F8-7	2.70–6.53 × 2.76–6.28
*B.* sp. 22B-F10-3	2.14–5.54 × 1.94–3.75
*B.* sp. 22B-F7-4	2.56–8.36 × 3.52–7.53
*B.* sp. 22B-F6-9	1.72–5.04 × 1.65–4.24
*B.* sp. 22B-F11-1	No sclerotia

A minimum of 10 sclerotia were measured per petri dish for each isolate. Measurement was done in two directions (length and width) of each sclerotia using a digital caliper.

Isolate 22B-F10-5 and 22B-F6-9 (Figures 3C,D) had sclerotia scattered in the fluffy mycelium. Isolates 22B-F11-1 (Figures 3E,F) and 22B-F9-3 produced no sclerotia after 14 days of incubation, and the underside of the culture plate appeared yellowish/orange. Isolates 22B-F8-1, 22B-F8-7 (Figures 3G,H), and 22B-F8-4 had medium to large-sized sclerotia, either solitary or aggregated in concentric rings. Isolates 22B-8-2, 22B-F6-10, 22B-F11-2, and 22B-F10-3 (Figures 3I,J) had fluffy and tufted mycelia with sclerotia in concentric rings. Isolate 22B-F7–4 had fluffy and tufted mycelia with large sclerotia (Table 1; Figures 3K,L).

#### 3.2.2 Phylogenetic identification of *Botrytis* species

To establish the identity of these isolates, partial DNA sequences of *ITS*, *G3PDH*, *HSP60*, and *RPB2* were subjected to phylogenetic analysis. The *ITS* sequence analysis for all the isolates corresponded to the genus *Botrytis*. Maximum parsimony *ITS* tree formed a polytomy (data not shown), which was not informative for distinguishing the different species. To confirm and distinguish the isolates, *G3PDH*, *HSP60*, and *RPB2* partial sequences were analyzed. The sequences from the Michigan isolates were aligned with sequences of *Botrytis* species retrieved from NCBI GenBank to generate phylogenetic trees for each gene and the concatenated gene (Supplementary Table S2). *Monilinia fructigena* and *Sclerotinia sclerotiorum* were used as outgroups in the phylogenetic analysis (Staats et al., 2005). The *G3PDH*, *HSP60*, and *RPB2* genes for 82, 79, and 78 isolates, respectively, were successfully amplified, and their sequences were included in phylogenetic analyses. Maximum likelihood (ML) and maximum parsimony (MP) phylogenetic trees showed some divergences in topology but similarity in species delimitation.

**Figure 4:**
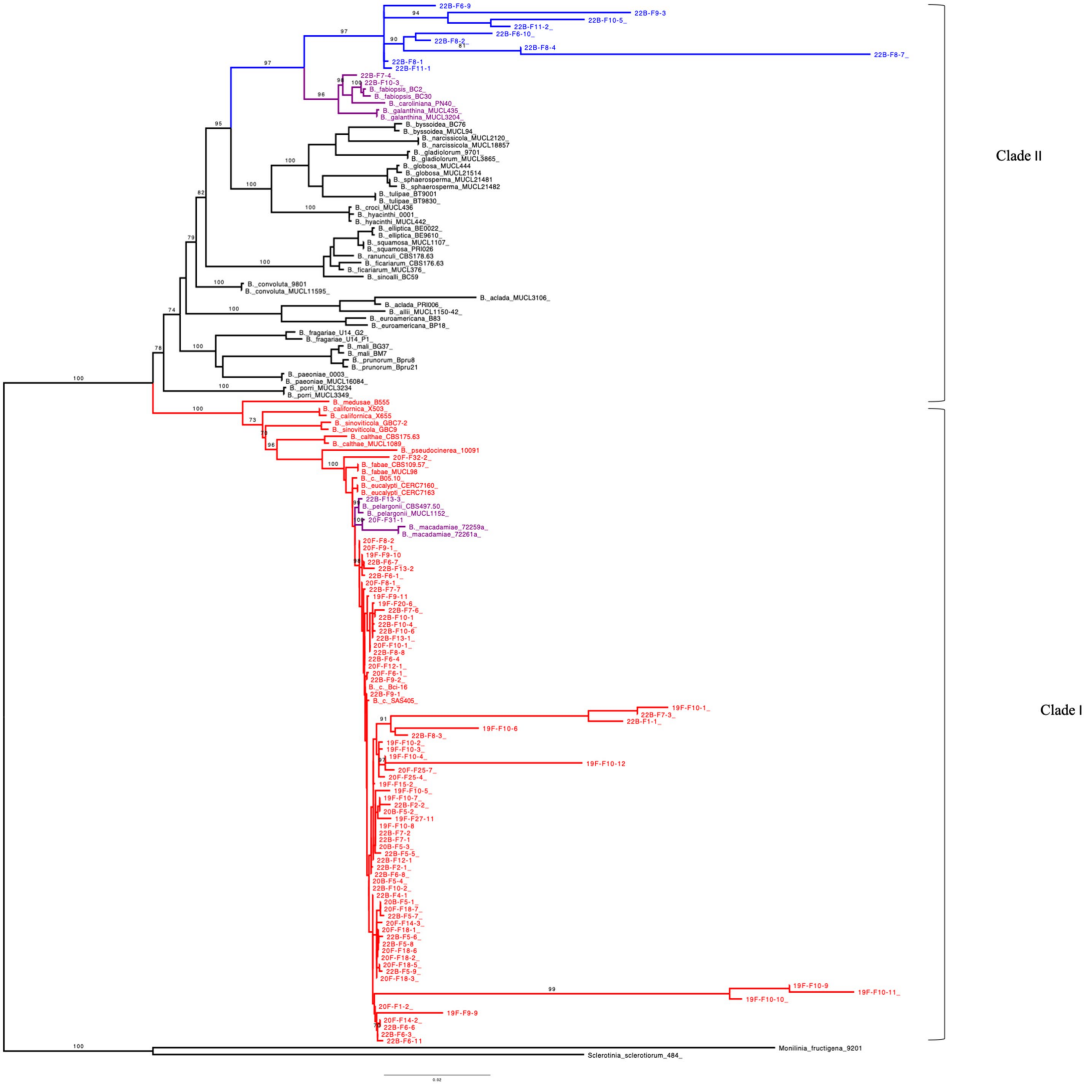
Maximum-likelihood tree inferred from 152 concatenated (*G3PDH + HSP60 + RPB2*) DNA sequences of *Botrytis* species, *Monilinia fructigena* and *Sclerotinia sclerotiorum*. Bootstrap values (BS) ≥ 70% (1,000 replicates) are shown. Number of parsimony informative sites: 659, Best-fit model according to BIC: TNe + R4, Number of constant sites: 2158, Number of distinct site patterns: 1685. Outgroups (*S. sclerotiorum* and *M. fructigena*) were used as roots. *Botrytis* species in Red represents clade I which contains *Botrytis cinerea* species complex (BCSC). *Botrytis* species in Blue denotes unidentified species in clade II and Purple represents undescribed species.

The ML tree was generated with the combined gene sequences (*G3PDH* + *HSP60* + *RPB2*) and grouped 86 of the Michigan *Botrytis* isolates into two clades (Figure 4). Seventy-four (74) of *Botrytis* isolates formed a well-supported cluster with *B. cinerea, B. fabae, B. pelargonii,* and *B. calthae, B. pseudocinerea*, *B. californica, B. sinoviticola, B. macademiae* and *B. medusae* (100% BS) in clade I. Ten isolates (22B-F9-3, 22B-F10-5, 22B-F6-9, 22B-F8-1, 22B-F11-1, 22B-F6-10, 22B-F8-2, 22B-F11-2, 22B-F8-4, and 22B-F8-7) formed a distinct and well supported (97% BS) subclade. Two isolates 22B-F7-4 and F10-3 clustered closely with *B. caroliniana*, *B. fabiopsis*, and *B. galanthina* (Figure 4).

In the ML trees obtained from *G3PDH*, *HSP60*, and *RPB2* sequences, most of Michigan *Botrytis* isolates formed a well-supported clade clustered with *B. cinerea* and its related reference sequences in clade I (> 95% BS) (Supplementary Figures S2–S4). In *G3PDH*, 11 isolates were contained in clade II. Eight of these isolates formed a subclade that is distantly related to *B. fabiopsis*, *B. caroliniana*, and *B. galanthina*. One isolate, 22B-F9-3, grouped with *B. euroamericana* (99% BS) (Supplementary Figure S2). In *RPB2*, nine (9) isolates formed a subclade that is related to *B. fabiopsis*, *B. caroliniana*, and *B. galanthina* (100% BS) (Supplementary Figure S3). For *HSP60*, the 74 isolates clustered with *B. cinerea* reference sequences. Isolates 22B-F7-4 and 22B-F10-3 clustered with *B. fabiopsis*, *B. caroliniana*, and *B. galanthina* (100% BS) (Supplementary Figure S4).

The MP tree generated from the combined sequences (G3PDH + HSP60 + RPB2) was similar to that of the ML (Supplementary Figure S5). Clade I contained 74 of the Michigan isolates clustered with *B. cinerea* reference sequences and its related cryptic species *B. fabae, B. pelargonii,* and *B. calthae, B. pseudocinerea*, *B. californica, B. sinoviticola, B. macademiae,* and *B. medusae* (99% BS). Within clade II, 10 of the Michigan isolates (22B-F9-3, 22B-F10-5, 22B-F6-9, 22B-F8-1, 22B-F11-1, 22B-F6-10, 22B-F8-2, 22B-F11-2, 22B-F8-4, 22B-F8-7) formed their own cluster. Similar to the trees from individual genes, two isolates, 22B-F7-4 and F10-3, were closely related to *B. caroliniana*, *B. fabiopsis*, and *B. galanthina* (Supplementary Figure S5).

In MP, the trees obtained from *G3PDH*, *HSP60*, and *RPB2* alignments formed a well-supported clade (> 80% BS). The *Botrytis* species were grouped into two main phylogenetic groups which were distantly related (clade I and clade II). Clade I contained most of the Michigan *Botrytis* isolates together with its related species. In the phylogenetic clade II, 12 Michigan *Botrytis* isolates (22B-F9-3, 22B-F10-5, 22B-F6-9, 22B-F8-1, 22B-F11-1, 22B-F6-10, 22B-F8-2, 22B-F11-2, 22B-F8-4, 22B-F8-7, and 22B-F7-4) clustered with *B. fabiopsis*, *B. caroliniana*, and *B. galanthina* in *G3PDH* and *RPB2*. Additionally, isolate 22B-F9-3 in *G3PDH* was closely related to *B. euroamericana* (Supplementary Figures S6, S7). In the *HSP60*, only two of the Michigan *Botrytis* isolates were in clade II (Supplementary Figure S8). All three of the trees revealed a close relationship between 22B-F7-4 and *B. caroliniana* and 22B-F10-3 and *B. fabiopsis* (Supplementary Figures S6–S8).

## 4 Discussion

*Botrytis cinerea* is an important pathogen of over 1,400 plant species (Elad et al., 2016). This necrotrophic pathogen is widespread, and it has been ranked second on the list of fungal pathogens based on their scientific and economic importance (Dean et al., 2012). Despite its importance and the extensive research done on the pathogen, *B. cinerea* remains one of the most challenging pathogens to manage due to its reproductive capability and tendency to develop resistance to multiple classes of fungicides. The emergence of fungicide resistance in *B. cinerea* populations raises significant concerns for growers of grapes, strawberries, blueberries, ornamentals, and some vegetable crops. Although prior research has characterized fungicide resistance within *B. cinerea* populations on grapevines in Michigan (Alzohairy et al., 2021), this study expands upon that knowledge by examining fungicide resistant *Botrytis* isolates from blueberries. The research findings describe *B. cinerea* isolates exhibiting diverse resistance to fungicides commonly used for botrytis blight control in Michigan. This highlights the potential for control failure of these classes of fungicides for disease management in blueberries.

Regardless of the year and region, the majority of *B. cinerea* isolates consistently showed high sensitivity to fludioxonil, fenhexamid, iprodione, fluopyram, and cyprodinil, whereas a significant proportion demonstrated lower sensitivity to thiabendazole, boscalid, and pyraclostrobin. Resistance frequencies for fungicides thiabendazole, boscalid, and pyraclostrobin were significantly higher in 2020 compared to 2019, and higher in the Southwest compared to the West region. However, this may be attributed to the bias in sampling. Given that similar fungicides and disease management practices are adopted in both regions, the sample sizes varied across the sampling years (2019, *n* = 131, 2020: *n* = 40, 2022: *n* = 37) and the two regions (Southwest: *n* = 150 and West: *n* = 58). Regardless of the sample size, the high resistance frequencies for fungicide such as pyraclostrobin suggests the need for continuous monitoring of pathogen populations. Although this study observed very low resistance frequency to some fungicides such as fenhexamid, other studies reported significantly high resistance frequency (Saito et al., 2016b; Amiri et al., 2018b). The variation in resistance frequency for such fungicides in other blueberry growing areas can be attributed to a combination of factors. Differences in historical fungicide use, genetic diversity of pathogen population, fungicide application practices and resistance management strategies employed by growers in different regions can significantly impact the rate of fungicide resistance development among pathogen (Hahn, 2014; Weber and Petridis, 2023).

This study demonstrates that fungicide resistance to pyraclostrobin, boscalid and thiabendazole among *B. cinerea* population are widespread in the blueberry growing areas of Michigan. The high number of isolates that were identified as resistant to pyraclostrobin, boscalid, cyprodinil, and thiabendazole in the 3 years of sampling suggests the accumulation of resistance to FRAC groups 11, 7, 9, and 1. These frequencies of resistant isolates could increase further if not managed properly. The observation of a high number of resistant isolates for these fungicides is not surprising as it has been reported frequently in literature (Alzohairy et al., 2021; Harper et al., 2021; Li, P. et al., 2023).

Multi fungicide resistance (MFR) has extensively been reported among *B. cinerea* populations in different crops (Alzohairy et al., 2021; Harper et al., 2021; Sofianos et al., 2023), hence it is not surprising that MFR was a recurring phenomenon in all 3 years. In this study, the phenotypes involving MFR to pyraclostrobin, boscalid, thiabendazole, and cyprodinil, and MFR to pyraclostrobin, boscalid, and thiabendazole were present in all years. The combination of pyraclostrobin, boscalid, and thiabendazole emerged as the most predominant MFR phenotypes, while pyraclostrobin and boscalid phenotypes were prevalent in 2022. For resistance management and disease control efficacy purposes, a number of these fungicides are co-formulated into a single product. For instance, boscalid is co-formulated with pyraclostrobin as Pristine® and cyprodinil is co-formulated with fludioxonil as Switch® (Syngenta, United States). The simultaneous resistance observed between pyraclostrobin and boscalid is expected because they are both respiration-inhibiting fungicides and in the same product. Additionally, fungicides with similar MOA are used to control other pathogens on blueberries fields. This leads to the exposure of the isolates to these fungicides regularly which could enhance the development of MFR. Furthermore, the study by Alzohairy et al. (2021) conducted on (Southwest) Michigan vineyards, revealed similar MFR pattern for phenotypes. Given this, it is also possible that fungal movement from other fields, especially from grape vineyards in the region, can contribute to the presence of these MFR isolates in blueberry fields. The emergence of MFR underscores the adaptability of *B. cinerea* populations, necessitating continuous surveillance and reassessment of fungicide management strategies. This consistency of MFR in all 3 years implies that the resistance is not a temporary phenomenon but rather an ongoing issue that requires attention.

This study also presents a first report on the identification and characterization of *Botrytis* species collected from blueberry fields in Michigan. The first attempt to phylogenetically classify *Botrytis* species was done by Staats et al. (2005) who divided 22 *Botrytis* species into two major groups, (clade I and clade II) using *ITS*, *G3PDH*, *HSP60,* and *RPB2* sequences. Clade I contained *B. cinerea*, *B. fabae, B. pelargonii,* and *B. calthae* and the other 18 species were in clade II. Following this study, several other species such as *B. pseudocinerea*, *B. californica,* and *B. sinoviticola, B. macademiae* and *B. medusae* have been added to clade I (Hyde et al., 2014; Saito et al., 2016a; Harper et al., 2019; Prasannath et al., 2021; Moparthi et al., 2023). Consistent with Staats et al. (2005), and previous studies, all the *Botrytis* species, based on the concatenated sequence were divided into two major groups, clade I and clade II. Most of the isolates were identified as part of the *B. cinerea* species complex (BCSC), thus they clustered with the species in Clade I with BS > 95%. Twelve isolates were positioned in clade II, BS > 78% which suggests that they could be undescribed *Botrytis* species in blueberry. The identification of these 12 isolates as undescribed or cryptic species is supported by the phylogenetic analyses of *G3PDH*, *HSP60,* and *RPB2* sequences, and the morphological and cultural characteristics of these isolates. Although *ITS* region was sequenced, the use of *ITS* alone is not sufficient to delineate *Botrytis* species and establish their taxonomic status (Staats et al., 2005). The phylogenetic tree inferred from *ITS* sequences resulted in a polytomy, which was not informative for distinguishing the different isolates. As a result, phylogenetic analysis of sequences from the three protein regions (*G3PDH*, *HSP60*, and *RPB2*) was used to delineate *Botrytis* species (Staats et al., 2005; Azevedo et al., 2020).

Within the BCSC, isolate 22B-F13-3 and 20F-F31-1 were closely related to *B. pelargonii* and *B. macademie*, respectively*. B. pelargonii* is closely related to *B. cinerea* but infects few crops such as pepper (*Capsicum annuum*), Ginseng (*Panax ginseng*) and Pelargonium (*Pelargonium grandiflorum*; Lu et al., 2019; Kazerooni et al., 2021; Fekrikohan et al., 2022). Staats et al. (2005), found that the internode separating *B. pelargonii* from *B. cinerea* was short; an indication of the high sequence similarity between the two species. *B. macademie* was reported on flowers of the macadamia tree (*Macadamia integrifolia*) and was closely related to *B. cinerea* (Prasannath et al., 2021, 2023). These two species have never been reported in blueberry or known to be associated with any *Vaccinium* species and as such could be a cryptic species in sympatry with *B. cinerea* in blueberry. This conclusion is drawn based on the phylogenetic position of isolates 22B-F13-3 and 20F-F31-1 on both the ML and MP trees from the concatenated sequences and the morphological features of these isolates. Also, isolates 19F-F27-6 and 22B-F32-2 were closely related to *B. fabae* and as such they could be *B. fabae.* The cultural characteristics of these two isolates were consistent with those described by Zhang et al. (2010) and Lee et al. (2020). These two species have not been reported in blueberry and as such this could be a cryptic species of the BCSC in blueberry.

The 12 isolates in clade II were grouped into two distantly related subclades. The first subclade contained two isolates (22B F10-3 and 22B F7-4) which are closely related to *B. fabiopsis*, *B. caroliniana*, and *B. galanthina* with BS > 90%. The morphological and cultural characteristics of 22B-F7–4 agree with descriptions of *B. caroliniana* described by Li et al. (2012) and Fernández-Ortuño et al. (2012). The characteristics of isolate 22B-F10-3 were similar to that of *B. fabiopsis* described by Li, Z. et al. (2023) and Bankina et al. (2021). Although the isolate 22B-F10-3 consistently grouped with *B. fabiosis,* with >96% BS for both concatenated and individual trees, sclerotia formation and size differed from the study by Li, Z. et al. (2023) and Zhang et al. (2010). Although Bankina et al. (2021) reported similar cultural characteristics, they did not record sclerotia formation. These differences could be due to intraspecific morphological characteristics or differences in incubating conditions. Based on their phylogenetic position and their morphological characteristics, isolates 22B F10-3 and 22B F7-4 can be described as *B. fabiopsis* and *B. caroliniana*, respectively. The 10 remaining isolates formed a well-supported (97% BS) second subclade which sustained themselves as a distinct *Botrytis* group. The closest species to these 10 isolates are *B. fabiopsis*, *B. caroliniana* and *B. galanthina*. While these isolates exhibited cultural and morphological features similar to *B. fabiopsis*, *B. caroliniana*, and *B. galanthina*, their distinct phylogenetic placements in both individual and concatenated trees identify them as a likely undescribed species or group of species. Some of these isolates also exhibited unique characteristics, including orange/yellow coloration of media and lack of sclerotia formation after 14 days (22B-F11-1 and 22B-F9-3). These isolates need further studies to establish their identity and pathogenicity. Aside from the genes used, the inclusion of necrosis and ethylene-inducing proteins (*NEP1* and *NEP2*) have been demonstrated to enhance species delineation in *Botrytis*. It will be essential to include these genes together with cultural and morphological characteristics of these isolates under different growth conditions in further analyses (Liu et al., 2016; Harper et al., 2019).

It is interesting to note that there was no difference among BCSC that were contained in clade I regarding the type of plant tissue from which they were isolated. This indicates that both blossom blight and fruit rot can be caused by *B. cinerea*. However, all the isolates (species) that grouped in clade II were isolated from blossoms. This could imply that this undescribed *Botrytis* species prefer or easily infect blossom tissue. It is worth noting that these undescribed species did not produce conidia in culture. Conidia are the fundamental means of dispersion for *B. cinerea* (Coertze et al., 2001), therefore the inability of a species to produce enough conidia throughout the season could result in less or no infections late in the season during fruiting. Additionally, the low proportion of these undescribed species in this study might be an indication of their fitness level and virulence, hence their inability to survive and reproduce until the end of the season to infect fruit.

The present study has shown several cases of fungicide resistance in *B. cinerea* isolates from some Michigan blueberry fields, showing different levels of single and multi-resistance to different fungicides. Data obtained from this study indicates that *B. cinerea* populations in Michigan have shifted toward resistance to pyraclostrobin, boscalid, and thiabendazole. *B. cinerea* isolates still have high sensitivity toward fludioxonil, fluopyram, and fenhexamid. The appearance of multiple fungicide resistance highlights the need for resistance management measures. Additionally, this study sheds light on the diversity of *Botrytis* populations within blueberry fields in Michigan. It reveals the presence of additional undescribed *Botrytis* species, including those belonging to the *B. cinerea* species complex, which were previously not reported in the state. Among these species, *B. cinerea* is the most prevalent. The findings presented mark the initial steps in characterizing a *Botrytis* spp. found in Michigan blueberries and provide valuable foundation for future investigations into this undescribed species.

## Data availability statement

The datasets presented in this study can be found in online repositories. The names of the repository/repositories and accession number(s) can be found in the article/Supplementary material.

## Author contributions

JA: Writing – original draft, Writing – review & editing. SA: Writing – review & editing. KN: Writing – review & editing. RH: Writing – review & editing. TM: Writing – review & editing.

## Footnotes

^1^RStudio PBC, Boston, MA. http://www.rstudio.com

^2^ https://www.ncbi.nlm.nih.gov
