# Supplementary material for: Fungicide resistance in Botrytis cinerea and identification of Botrytis species associated with blueberry in Michigan
Source: Front Microbiol. 2024 Jul 22;15:1425392. doi: 10.3389/fmicb.2024.1425392 (PMC11298438; doi:10.3389/fmicb.2024.1425392)
Supplement: Supplementary file 1 [file Data_Sheet_1.docx]

**Supplementary Material**

Supplementary Figure 1. Frequencies of fungicide resistance in *Botrytis cinerea* collected from blossoms and fruit of blueberries grown in Southwest and West Michigan. The number of isolates was 150 and 58, respectively. Bars with “*****” denote a significantly higher (*P* < 0.05) resistance frequency between Southwest and West Michigan.

Supplementary Figure 2. Maximum-likelihood tree inferred from 152 partial DNA sequences of *G3PDH* of *Botrytis* species, *Monilinia fructigena* and *Sclerotinia sclerotiorum*. Bootstrap values (BS) ≥ 70 % (1000 replicates) are shown. Number of parsimony informative sites: 169, Best-fit model according to BIC: TNe+R3, Number of constant sites: 735, Number of distinct site patterns: 340. Outgroups (*S. sclerotiorum* and *M. fructigena*) were used as roots. *Botrytis* species in Red represents clade I which contains *Botrytis cinerea* species complex (BCSC). *Botrytis* species in Blue denotes unidentified species in clade II and Purple represents newly identified species.

Supplementary Figure 3. Maximum-likelihood tree inferred from 150 partial DNA sequences of *RPB2* of *Botrytis* species, *Monilinia fructigena* and *Sclerotinia sclerotiorum*. Bootstrap values (BS) ≥ 70 % (1000 replicates) are shown. Number of parsimony informative sites: 220, Best-fit model according to BIC: TIM2e+G4, Number of constant sites: 854, Number of distinct site patterns: 376. Outgroups (*S. sclerotiorum* and *M. fructigena*) were used as roots. *Botrytis* species in Red represents clade I which contains *Botrytis cinerea* species complex (BCSC). *Botrytis* species in Blue denotes unidentified species in clade II and Purple represents newly identified species.

Numb Number of distinct site patterns: 340

Number of distinct site patterns: 340est-fit model according to BIC: TNe+R3

Supplementary Figure 4. Maximum-likelihood tree inferred from 151 partial DNA sequences of *HSP60* of *Botrytis* species, *Monilinia fructigena* and *Sclerotinia sclerotiorum*. Bootstrap values (BS) ≥ 70 % (1000 replicates) are shown. Number of parsimony informative sites: 211, Best-fit model according to BIC: TNe+G4, Number of constant sites: 735, Number of distinct site patterns: 351. Outgroups (*S. sclerotiorum* and *M. fructigena*) were used as roots. *Botrytis* species in Red represents clade I which contains *Botrytis cinerea* species complex (BCSC). *Botrytis* species in Blue denotes unidentified species in clade II and Purple represents newly identified species.

Supplementary Figure 5. Maximum parsimony tree inferred from 152 concatenated (*G3PDH + HSP60 + RPB2*) DNA sequences of *Botrytis* species, *Monilinia fructigena* and *Sclerotinia sclerotiorum*. Bootstrap values (BS) ≥ 70 % (1000 replicates) are shown. The consistency index is 0.641656, the retention index is 0.894154, and the composite index is 0.573739. Outgroups (*Sclerotinia sclerotiorum* and *Monilinia fructigena*) were used as roots. *Botrytis* species in Red represents clade I which contains *Botrytis cinerea* species complex (BCSC). *Botrytis* species in Blue denotes unidentified species in clade II and Purple represents newly identified species.

Supplementary Figure 6. Maximum parsimony tree inferred from 152 partial DNA sequences of *G3PDH* of *Botrytis* species, *Monilinia fructigena* and *Sclerotinia sclerotiorum*. Bootstrap values (BS) ≥ 70 % (1000 replicates) are shown. The consistency index is 0.677778, the retention index is 0.928324, and the composite index is 0.629198. Outgroups (*S. sclerotiorum* and *M. fructigena*) were used as roots. *Botrytis* species in Red represents clade I which contains *Botrytis cinerea* species complex (BCSC). *Botrytis* species in Blue denotes unidentified species in clade II and Purple represents newly identified species.

Supplementary Figure 7. Maximum parsimony tree inferred from 150 partial DNA sequences of *RPB2* of *Botrytis* species, *Monilinia fructigena* and *Sclerotinia sclerotiorum*. Bootstrap values (BS) ≥ 70 % (1000 replicates) are shown. The consistency index is 0.683656, the retention index is 0.946301, and the composite index is 0.646944. Outgroups (*S. sclerotiorum* and *M. fructigena*) were used as roots. *Botrytis* species in Red represents clade I which contains *Botrytis cinerea* species complex (BCSC). *Botrytis* species in Blue denotes unidentified species in clade II and Purple represents newly identified species.

Supplementary Figure 8. Maximum parsimony tree inferred from 151 partial DNA sequences of *HSP60* of *Botrytis* species, *Monilinia fructigena* and *Sclerotinia sclerotiorum*. Bootstrap values (BS) ≥ 70 % (1000 replicates) are shown. The consistency index is 0.671304, the retention index is 0.925296, and the composite index is 0.621156. Outgroups (*S. sclerotiorum* and *M. fructigena*) were used as roots. *Botrytis* species in Red represents clade I which contains *Botrytis cinerea* species complex (BCSC). *Botrytis* species in Blue denotes unidentified species in clade II and Purple represents newly identified species.

.

Supplemental Table 1. List of amplification and sequencing primers for the identification of *Botrytis cinerea*

| Primer name^A^ | Primer sequence (5’ – 3’)^B^ | Originated from |
| --- | --- | --- |
| **ITS** |  |  |
| ITS1+ | gtgactgtaaaacgacggccagtTCCGTAGGTGAACCTGCGG | Staats et al., 2005 |
| ITS4+ | gtgaccaggaaacagctatgaccTCCTCCGCTTATTGATATGC | Staats et al., 2005 |
| **G3PDH** |  |  |
| G3PDHfor+ | gtgactgtaaaacgacggccagtATTGACATCGTCGCTGTCAACGA | Staats et al., 2005 |
| G3PDHrev+ | gtgaccaggaaacagctatgaccACCCCACTCGTTGTCGTACCA | Staats et al., 2005 |
| **HSP60** |  |  |
| HSP60for+ | gtgactgtaaaacgacggccagtCAACAATTGAGATTTGCCCACAAG | Staats et al., 2005 |
| HSP60rev+ | gtgaccaggaaacagctatgaccGATGGATCCAGTGGTACCGAGCAT | Staats et al., 2005 |
| **RPB** |  |  |
| RPB2for+ | gtgactgtaaaacgacggccagtGATGATCGTGATCATTTCGG | Staats et al., 2005 |
| RPB2rev+ | gtgaccaggaaacagctatgaccCCCATAGCTTGCTTACCCAT | Staats et al., 2005 |
| ^A^ Bold text indicates primer target.  ^B^ Lower-case letters denote a primer extension with M13(-20) forward primers or M13 reverse primers. | | |

Supplemental Table 2. List of *Botrytis* species and isolates, *Monilinia* and *Sclerotinia* and their GenBank Accession numbers for DNA sequences used as reference (https://www.ncbi.nlm.nih.gov)

| **Isolate Names** | **Collection number** | GenBank Accession numbers of DNA sequences | | | |
| --- | --- | --- | --- | --- | --- |
|  |  | ***G3PDH*** | ***HSP60*** | ***RPB2*** | ***ITS*** |
| *B. aclada* | MUCL3106 | AJ704991 | AJ716049 | AJ745663 | * |
|  | MUCL8415 | AJ704992 | AJ716050 | AJ745664 | * |
|  | PRI006 | AJ704993 | AJ716051 | AJ745665 | AJ716295 |
| *B. allii* | MUCL1150-42 | AJ704994 | * | * | * |
|  | MUCl1150-31 | * | AJ716055 | * | * |
|  | MUCL1150-122 | * | * | AJ745668 | * |
|  | OTU3323 | * | * | * | MT595943 |
| *B. anthophila* | CBS122.26 | * | * | * | AJ716305 |
| *B. byssoidea* | BC76 | FJ169652 | FJ169661 | FJ169681 | FJ169671 |
|  | MUCL94 | AJ704998 | AJ716059 | AJ745670 | * |
|  | HSAUP074125 | * | * | * | FJ914712 |
| *B. cinerea* | B05.10 | MT360701 | AJ716063 | AJ745674 | AM235297 |
|  | Bci-16 | KP120864 | KP120871 | KP136776 | KP091847 |
|  | SAS405 | AJ705005 | AJ716066 | AJ745678 | * |
| *B. californica* | X503 | KJ937068 | KJ937058 | KJ937048 | KJ937038 |
|  | X655 | KJ937069 | KJ937059 | KJ937049 | KJ937039 |
| *B. calthae* | CBS175.63 | AJ704999 | AJ716060 | AJ745671 | AJ716302 |
|  | MUCL1089 | AJ705000 | AJ716061 | AJ745672 | * |
| *B. caroliniana* | PN40 | OP019701 | OP019699 | ON887326 | ON807238 |
| *B. convoluta* | 9801 | AJ705007 | AJ716068 | AJ745679 | AJ716304 |
|  | MUCL11595 | AJ705008 | AJ716069 | AJ745680 | * |
|  | CBS427.68 | * | * | * | MH859173 |
| *B. croci* | MUCL436 | AJ705009 | AJ716070 | AJ745681 | * |
| *B. deweyae* | 5-Apr | OQ164791 | * | * | * |
|  | CBS134650 | * | * | * | NR_171711 |
| *B. draytonii* | CBS613.72 | * | * | * | MH860596 |
| *B. elliptica* | BE0022 | AJ705010 | AJ716071 | AJ745682 | AM235307 |
|  | BE9610 | AJ705011 | AJ716072 | AJ745683 | AM235302 |
| *B. eucalypti* | CERC7160 | KX301018 | KX301022 | KX301026 | KX301014 |
|  | CERC7163 | KX301019 | KX301023 | KX301027 | KX301015 |
| *B. euroamericana* | B83 | KC191677 | KC191678 | KC191679 | KC191680 |
|  | BP18 | KY200364 | KY203867 | KY211625 | * |
| *B. fabae* | BF12 | OR659436 | OR659435 | OR659437 | OR647512 |
|  | CBS109.57 | AJ705013 | AJ716074 | AJ745685 | AJ716303 |
|  | MUCL98 | AJ705014 | AJ716075 | AJ745686 | * |
| *B. fabiopsis* | BC2 | EU519211 | EU514482 | EU514473 | EU519204 |
|  | BC30 | EU563106 | EU563097 | EU563117 | EU563126 |
|  | BC13 | * | * | * | EU563122 |
| *B. ficariarum* | CBS176.63 | AJ705015 | AJ716076 | AJ745687 | AJ716296 |
|  | MUCL376 | AJ705016 | AJ716077 | AJ745688 | * |
| *B. fragariae* | U14_G2 | KX429700 | KX429693 | KX429707 | * |
|  | U14_P1 | KX429699 | KX429692 | KX429706 | * |
| *B. galanthina* | MUCL435 | AJ705018 | AJ716079 | AJ745689 | * |
|  | MUCL3204 | AJ705017 | AJ716078 | AJ745690 | * |
|  | CBS127.37 | * | * | * | MH855851 |
|  | CBS138.60 | * | * | * | MH857925 |
| *B. gladiolorum* | 9701 | AJ705019 | AJ716080 | AJ745691 | * |
|  | MUCL3865 | AJ705020 | AJ716081 | AJ745692 | * |
| *B. globosa* | CBS388.52 | * | * | * | MH857092 |
|  | MUCL444 | AJ705022 | AJ716083 | AJ745693 | * |
|  | MUCL21514 | AJ705021 | AJ716082 | AJ745694 | * |
| *B. hyacinthi* | 001 | AJ705023 | AJ716084 | AJ745695 | * |
|  | MUCL442 | AJ705024 | AJ716085 | AJ745696 | * |
|  | 1471069 | * | * | * | FJ710809 |
| *B. macadamiae* | 72259a | MZ344223 | MZ344234 | MZ356230 | * |
|  | 72261a | MZ344224 | MZ344235 | MZ356231 | * |
| *B. mali* | BG37 | MT604432 | MT604532 | MT604336 | * |
|  | BM7 | MT604433 | MT604533 | MT604337 | * |
| *B. medusae* | B555 | MH732861 | MH732866 | MH732870 | * |
| *B. narcissicola* | MUCL2120 | AJ705026 | AJ716087 | AJ745697 | * |
|  | MUCL18857 | AJ705025 | AJ716086 | AJ745698 | * |
|  | F105830 | * | * | * | KJ941071 |
| *B. paeoniae* | 003 | AJ705027 | AJ716088 | AJ745699 | AJ716298 |
|  | MUCL16084 | AJ705028 | AJ716089 | AJ745700 | * |
|  | CBS127.58 | * | * | * | MH857720 |
| *B. pelargonii* | CBS497.50 | AJ704990 | AJ716046 | AJ745662 | MH856724 |
|  | MUCL1152 | AJ705029 | AJ716090 | AJ745701 | * |
|  | SR-MH11 | KX987154 | KX987155 | KX987156 | KX987153 |
| *B. polyblastis* | CBS287.38 | * | AJ716091 | AJ745702 | AJ716291 |
|  | MUCL21492 | * | AJ716092 | AJ745703 | * |
| *B. polygoni* | Poly-1 | MG846496 | MG846501 | MG846506 | * |
|  | Poly-2 | MG846497 | MG846502 | MG846507 | * |
| *B. porri* | MUCL3234 | AJ705032 | AJ716093 | AJ745704 | AJ716292 |
|  | MUCL3349 | AJ705033 | AJ716094 | AJ745705 | * |
|  | 80339 | * | * | * | OR761544 |
| *B. prunorum* | Bpru8 | KP339979 | KP339993 | KP339986 | KP234035 |
|  | Bpru21 | KP339980 | KP339994 | KP339987 | KP234036 |
| *B. pseudocinerea* | 10091 | JN692414 | JN692400 | JN692428 | JN692379 |
| *B. ranunculi* | CBS178.63 | AJ705034 | AJ716095 | AJ745706 | MH869860 |
| *B. sinoalli* | BC59 | FJ169646 | FJ169658 | FJ169678 | FJ169664 |
|  | BC23 | * | * | * | EU519203 |
| *B. sinoviticola* | GBC7-2 | JN692406 | FJ169658 | FJ169678 | JN692376 |
|  | GBC9 | JN692408 | JN692392 | JN692420 | JN692378 |
| *B. sphaerosperma* | MUCL21481 | AJ705035 | AJ716096 | AJ745708 | AJ716293 |
|  | MUCL21482 | AJ705036 | AJ716097 | AJ745709 | * |
| *B. squamosa* | MUCL1107 | AJ705037 | AJ716098 | AJ745710 | * |
|  | PRI026 | AJ705039 | AJ716100 | AJ745707 | AJ716299 |
|  | BC2 | * | * | * | FJ169668 |
| *B. tulipae* | BT9001 | AJ705040 | AJ716101 | AJ745712 | AM235308 |
|  | BT9830 | AJ705041 | AJ716102 | AJ745713 | AJ716301 |
| *Monilinia fructigena* | 9201 | AJ705043 | AJ716047 | AJ745715 | * |
|  | 2319 | * | * | * | AB470882 |
| *Sclerotinia sclerotiorum* | 484 | AJ705044 | AJ716048 | AJ745716 | * |
|  | CBS499.50 | * | * | * | KF859933 |
